# Respiratory distress and chest pain: a perforated peptic ulcer with an unusual presentation

**DOI:** 10.1186/1865-1380-4-34

**Published:** 2011-06-22

**Authors:** David I Bruner, Corey Gustafson

**Affiliations:** 1 Uniformed Services University of the Health Sciences, Bethesda, MD, USA; 2 Emergency Department, Naval Medical Center Portsmouth, Portsmouth, VA, USA

## Abstract

**Background:**

Dyspnea and chest pain are common presenting complaints to the ED, and coupled together can present a challenging diagnostic dilemma in patients in extremis. A thoughtful evaluation is required, giving due diligence to the immediate life threats as well as multiple etiologies which can cause serious morbidity. A perforated peptic ulcer is one such possibility and requires rapid diagnosis and prompt intervention to avoid the associated high risk of morbidity and mortality.

**Method:**

We present a case report of a 54 year old man with respiratory distress and chest pain as the initial Emergency Department presentation of a perforated duodenal ulcer.

**Results:**

We discuss an unusual presentation of a perforated duodenal ulcer that was recognized in the emergency department and treated promptly. The patient was surgically treated immediately, had a prolonged and complicated post-operative course, but is ultimately doing well. We also provide a brief literature review of the risk factors, imaging choices, and management decision required to treat a perforated ulcer.

**Conclusions:**

Perforated ulcers can have highly varied presentations and are occasionally difficult to diagnose in a complicated patient. Knowledge of the risk factors and a thorough history and physical can point to the diagnosis, but timely and appropriate imaging is often required because delays in diagnosis and treatment lead to poor outcomes. Early administration of antibiotics and immediate surgical repair are necessary to limit morbidity and mortality.

## Introduction

Dyspnea and chest pain are common presenting complaints to the Emergency Department (ED), and they often occur concurrently. This combination of symptoms presents a diagnostic challenge for any physician given the broad differential each complaint entails. A thoughtful and judicious workup is required, and avoidance of anchoring on a particular diagnosis is necessary to avoid missing alternative, equally life-threatening possibilities. We present the case of a patient with perforated duodenal ulcer who initially arrived with respiratory distress and hypoxia.

## Case presentation

A 54-year-old white male presented to the Emergency Department with complaints of progressive dyspnea and chest pain that had started simultaneously with acute onset 10 h before arrival. He stated the chest pain started while going from a seated to standing position. The pain was substernal and sharp with epigastric radiation initally. The pain was also noted to be worse with movement, and although it was still present, it had subsequently waned since the initial symptom onset. His dyspnea started immediately after the onset of chest pain and was worse with exertion. At presentation, he had progressed to the point of breathlessness, prompting his ED visit. Review of systems revealed no nausea, vomiting, diarrhea, fevers, or recent cough or congestion, as well as no similar episodes of pain or history of coronary artery disease, heart failure, chronic obstructive pulmonary disease, gastro-esophageal reflux disease, or GI bleeding episodes.

His past medical history was significant for osteoarthritis and benign prostatic hypertrophy, and he denied any prior surgery. His medications included ibuprofen (800 mg three times a day with meals), which he has taken routinely over the past month. Of note, he had smoked a pack of cigarettes per day for the past 40 years and claimed only occasional alcohol usage.

Physical examination revealed an obese, ashen colored male in obvious respiratory distress. Vital signs were temperature of 36.4°C (97.5°F), heart rate 118, respiratory rate 36, oxygen saturation 77% on room air, and blood pressure 151/88 mmHg. The patient was alert, oriented and in obvious discomfort. His cardiovascular examination was remarkable for tachycardia, with regular and strong distal pulses in all four extremities. Pulmonary evaluation demonstrated clear breath sounds in the upper and lower lung fields, with diminished volume in the bases. His abdomen was soft and mildly distended with slight but diffuse tenderness to soft touch and percussion without tympany or guarding. Stool was positive for occult blood. A bedside abdominal ultrasound was performed and was negative for free fluid or abdominal aortic aneurysm. The ultrasound was difficult to perform because the patient became increasingly dyspneic and anxious while laying supine and was unable to lay still. His skin was ashen and diaphoretic without petechiae, purpura, or stigmata of liver disease.

**Figure 1:**
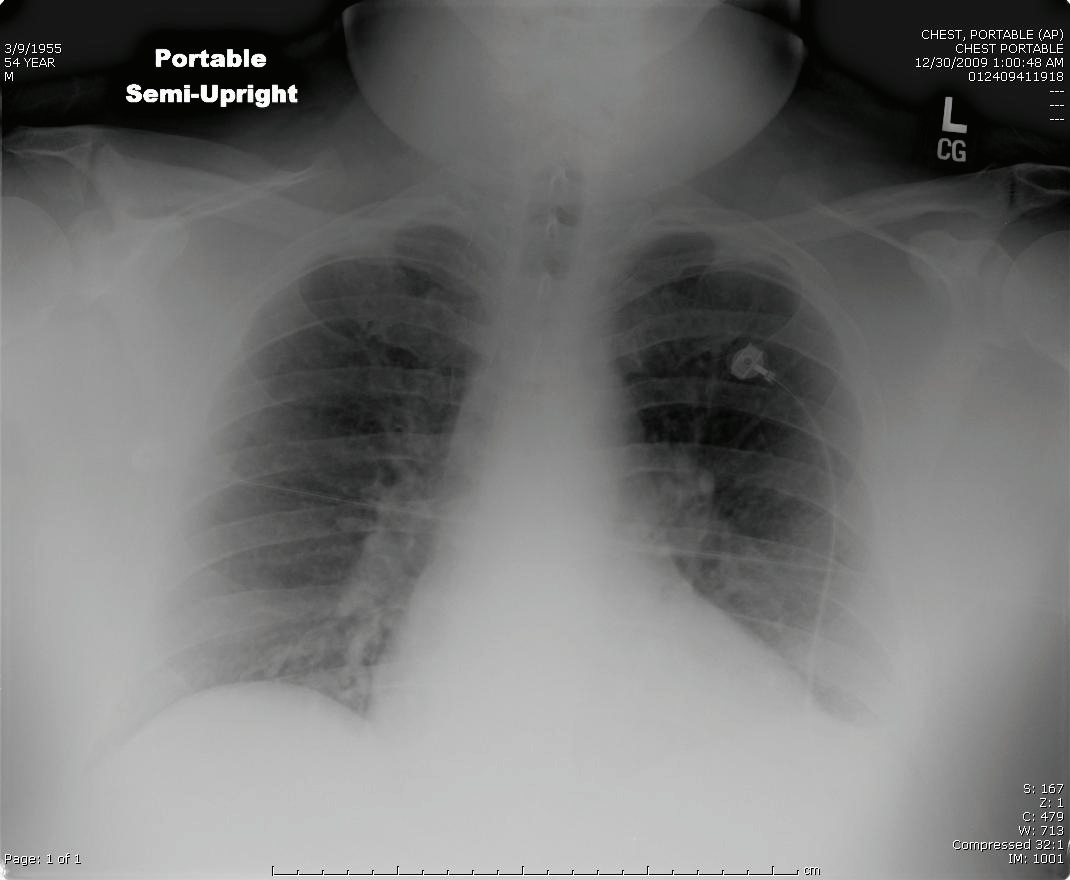
**Upright portable chest X-ray**. No acute cardiopulmonary process was noted and no intra-abdominal free air.

Initial diagnostics ordered included an electrocardiogram revealing sinus tachycardia and no ischemic changes, and an upright portable chest x-ray (see Figure 1) that was unremarkable for acute cardiopulmonary processes or free air in the abdomen. Laboratory analysis showed an elevated i-stat troponin-I of 0.74 ng/ml (normal <0.034 ng/ml), D-dimer was 5.73 mcg/ml (normal <0.48 mcg/ml), and a white blood cell count of 18.8 (× 1,000/ul) with a left shift. Electrolytes, renal function, and coagulation studies were normal, and his lactate was 1.4 mmol/l (normal <2.2 mmol/l).

He was immediately started on oxygen using a non-rebreather with immediate improvement in his dyspnea and oxygen saturation. His pain was addressed using morphine. Given his positive troponin, 162 mg of aspirin was given; heparin was withheld because the patient was guaiac positive. Shortly after his improved chest pain and respiratory distress, the patient stated that the abdominal pain was more prominent.

**Figure 2:**
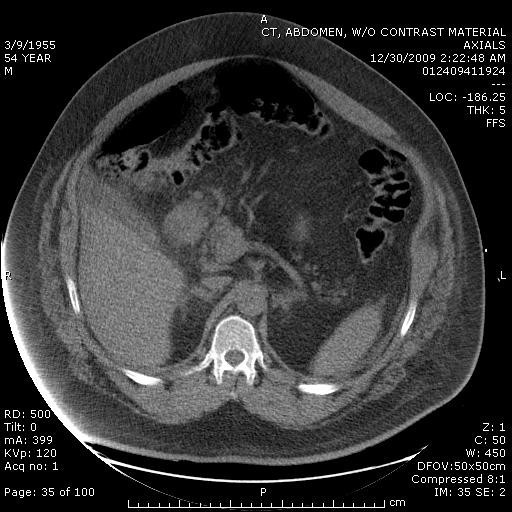
**Non-contrast CT of abdomen revealing intra-abdominal free air and perforated viscus**.

Cardiology consultation was considered because of the troponin elevation, but because of the increased abdominal pain, a non-contrasted CT of the abdomen (see Figure 2) was obtained, which revealed free air in the abdomen and a perforated duodenal ulcer.

Intravenous fluid resuscitation, antibiotics, and proton-pump inhibitor therapy were ordered, and surgery was consulted. He was taken to the operating room for definitive treatment with an omental patch procedure. His hospital stay was long and complicated by an ischemic stroke, but he was eventually discharged to a rehabilitation facility and is expected to have a functional recovery.

## Discussion

Acute bowel perforations are potentially life-threatening events that must be recognized immediately in order to begin prompt treatment and surgical intervention because of the high risk of morbidity and mortality if not recognized in a timely manner. This case is unique because there has been only one reported duodenal ulcer perforation patient to present with hypoxia and dyspnea as initial symptoms [1]. This discussion focuses primarily on the diagnosis and ED management of peptic and duodenal ulcer perforations.

### Epidemiology and risk factors

Perforated duodenal ulcers typically occur in patients with known peptic ulcer disease (PUD). PUD in the United States is most commonly due to *Helicobacter pylori (H. pylori) *or non-steroidal anti-inflammatory drug (NSAID) use. The estimated rate of perforation or bleeding in patients with known peptic ulcer disease is 1-2% per year. Duodenal ulcers are associated with 60% of perforations due to peptic ulcer disease. In contrast, antral and gastric body ulcers each account for 20% of perforated ulcers. NSAID use is associated with up to one-half of perforated ulcers [2,3]. Smoking, age over 65, and a history of complicated ulcer disease are also associated with a higher risk of ulcer perforation.

Our patient's only risk factor for a duodenal ulcer was his frequent use of NSAIDs for his arthritis. According to the Food and Drug Administration, NSAIDs are associated with a 1-4% risk per year of significant gastrointestinal events, accounting for 3,000 deaths per year, and the risk of complications is related to the daily dose of NSAIDs ingested [4]. NSAIDs have excellent utility in providing analgesia for a variety of conditions, but they are known to cause injury to the gastric and duodenal mucosa, leading to ulcer formation, bleeding, and possible perforation. These medications are taken daily in the US by approximately 3 million people, and approximately 10% of people on daily NSAIDs will have an acute ulcer [4].

NSAIDs are more likely to produce gastric ulcers rather than duodenal ulcers, but they are known to cause duodenal ulcers as well. Lanas et al. demonstrated that the use of NSAIDs increased the risk of bleeding from a peptic ulcer with an odds ratio of 7.4 [5]. Smedley and colleagues, however, showed that NSAID use was only associated with 12% of duodenal ulcer perforations and 13% of duodenal ulcer bleeding. They also reported several older studies with similar results [6].

*Helicobacter pylori *has been shown to be the cause of duodenal ulcers in up to 61% of patients [7]. *H. pylori *is the most common known cause of peptic and duodenal ulcer disease. It is estimated that up to 90% of duodenal ulcers and 75% of gastric ulcers are due to *H. pylori *infection [4]. The incidence of *H. pylori *appears to be decreasing in frequency in developed nations because of changes in diet, increased use of proton-pump inhibitors, and improved personal hygiene over the last few decades, but it remains a significant cause of PUD in the older population [8]. The association between *H. pylori *and perforation is unclear, however, with some studies finding a significant relationship and others suggesting minimal to no association, which suggests that chronic ulcer disease has a different pathophysiology from acute duodenal ulcer perforation [9,10].

### Clinical presentation

The history of present illness in patients with perforated ulcers frequently reflects a history of PUD, but many patients will deny the diagnosis of PUD despite a prior history of indigestion symptoms. Typically, initial symptoms begin with an onset of severe abdominal pain that is commonly epigastric in location, but becomes generalized as a chemical peritonitis ensues. This is often associated with vomiting, diaphoresis, and an ashen appearance in early stages. Temperatures may frequently be subnormal [11]. The pain may begin to subside within several hours, leading some to suspect they are improving.

Cope's textbook of surgery reports three phases of presentation for perforated ulcers [11]. Phase one consists of the pain as noted above, which is when most patients will present to care because of the severity of pain. Phase two occurs between 2 and 12 h from symptom onset, and pain will often improve during this time. However, the patient will most likely have a persistently rigid abdomen, pain with movement, spreading of pain to include the lower quadrants as fluid and air fill the abdomen, and shallow respirations. Phase three (12 h and beyond) is associated with abdominal distention, generalized peritonitis, and hemodynamic collapse, which occurs in approximately 5-10% of patients, most often in those between the ages of 40 and 60 years old [12]. Early recognition of this disease is essential because the overall prognosis is good if managed within the first 6 h of perforation, whereas mortality is much higher if there is a delay in diagnosis or presentation of greater than 12 h [11,13].

Our patient most likely presented during phase two of this disease process as his pain had improved, but he was having respiratory distress, which may have over-taxed his myocardium causing a troponin leak. It is also possible that our patient's respiratory distress may have been secondary to myocardial dysfunction. We believed that his troponin elevation, myocardial dysfunction, and subsequent respiratory symptoms were likely secondary to the overall systemic inflammatory response resulting from the perforated ulcer, thus increasing myocardial oxygen demand to a level that his heart could not match.

### Diagnostic imaging

Imaging choices for diagnosing bowel perforations include plain films and computed tomography. An upright chest x-ray is an excellent first choice. A positive upright chest x-ray (free air beneath the diaphragm) can acutely make the diagnosis, but plain films can miss 15% to 30% of patients with free air in the abdomen according to surgical texts [4,11]. Some authors suggest insufflation of 200 to 300 ml of air via a nasogastric tube to increase the yield of plain films, but they offer no data as to how much this may help [14]. Specifically for duodenal ulcers, 10-20% of patients will not have free air on plain films.

If a CT is performed with contrast, one should use water-soluble gastrograffin contrast. A leak of contrast confirms the diagnosis. Small studies have examined this and suggested that CT is 100% sensitive in the diagnosis of pneumoperitoneum, whereas upright chest film was only 33% sensitive for small pockets of air [15]. No studies have compared contrast versus non-contrast CT for this disease, but both are capable of making an accurate diagnosis [10,11,16]. In a small minority of patients with perforated duodenal ulcers, there will be no free air, and only free fluid will be present on CT [16].

A non-contrast CT scan was obtained in our patient for the sake of expediency as we did not feel a 2-h delay (the standard requirement for contrast CT scans at our institution) would be appropriate for a patient with a potentially perforated ulcer with significant tachycardia and hypoxia. Because we were also considering acute myocardial infarction as the cause of his symptoms, we did not want to delay appropriate cardiology consultation and treatment if no intra-abdominal pathology were found.

### Management and prognosis

Acute management of these patients in the emergency department involves several different steps, but most importantly, the diagnosis must be made quickly, and general surgeons should be involved immediately upon making the diagnosis of a perforated ulcer.

There are no exact recommendations for pain control, but adequate pain control with opioid medications should be initiated promptly with consideration for the patient's hemodynamic status. There are multiple studies showing that opioid medications do not mask peritonitis in other surgical cases such as appendicitis and cholecystitis, and it seems unlikely that pain control will mask the peritonitis of a bowel perforation [17-20].

Initiation of treatment to reduce acid secretion with proton pump inhibitors should also be started to try to decrease spillage of acidic fluid into the abdomen. Also, broad spectrum antibiotics should be started upon recognition of a bowel perforation. Antibiotic choices include piperacillin/tazobactam, cefotaxime, amoxicillin, or a flouroquinolone plus metronidazole. There should be no delay in the administration of antibiotics. Studies have suggested that up to 13% of patients receive inappropriate initial antibiotics, which may lead to a worse prognosis [13,21].

Emergent surgical consultation is required for operative repair of the site of bowel perforation. Clearly, the source of the perforation will determine the type and extent of surgery, but the majority of these are managed with an omental patch closure with or without a parietal cell or truncal vagotomy.

## Conclusion

Our patient's presentation was particularly unusual because the presenting signs and symptoms were those of chest pain and respiratory distress. As those symptoms improved, the patient subsequently had acute worsening of abdominal pain prompting the CT scan that yielded the diagnosis of a perforated duodenal ulcer. Considering the patient had significant abdominal girth, his pain seemed minimal on exam, and the acute peritonitis and free air in the peritoneal cavity may have irritated and elevated his diaphragm enough to cause chest pain, shortness of breath, and hypoxia. He also had an elevated troponin, suggesting that the stress of his bowel perforation and systemic illness taxed his myocardium, causing the troponin leak and subsequent decreased cardiac output resulting in hypoxia.

## Consent

Written informed consent was obtained from the patient for publication of this case report and any accompanying images. A copy of the written consent is available for review by the Editor-in-Chief of this journal.

## Abbreviations

ED: emergency department; CT: computed tomography; NSAID: non-steroidal anti-inflammatory drug; PUD: peptic ulcer disease.

## Competing interests

The authors declare that they have no competing interests.

## Authors' contributions

DB conceived the idea for this case report, performed the literature review, wrote the case discussion, and provided formatting for the article. CG assisted with the literature review and wrote the case report section of this manuscript. All authors read and approved the final manuscript.
